# Chloroplast genome of *Corydalis impatiens* (Pall.) Fisch. ex DC. (Papaveraceae), a Tibetan medical herb

**DOI:** 10.1080/23802359.2022.2104668

**Published:** 2022-08-01

**Authors:** Digao Wan, Haijuan Bao, Qupei Danzeng, Xiao Guo, Qien Li

**Affiliations:** a Tibetan Medicine Research Center, Tibetan Medical College, Qinghai University, Xining, People’s Republic of China; b College of Ecological Environment and Resources, Qinghai Minzu University, Xining, People’s Republic of China; c University Tibetan Medicine, Lhasa, People’s Republic of China

**Keywords:** Chloroplast genome, *Corydalis impatiens* (Pall.) Fisch 1821, evolutionary analysis, Papaveraceae, Tibetan medical herb

## Abstract

*Corydalis impatiens* (Pall.) Fisch. 1821. (Papaveraceae) is a Tibetan medical herb used to reduce pain, treat skin injuries, cure hepatitis, and benefit the circulatory system. In the current study, the chloroplast genome of *C. impatiens* was sequenced. This complete genome is a circular 197,317 bp sequence consisting of a small single-copy (SSC, 3105 bp) region, a large single-copy (LSC, 89,790 bp) region, and a pair of inverted repeats (IRs, 52,211 bp). This chloroplast genome encodes a total of 127 functional genes, including 81 protein-coding, 38 transfer RNA, and eight ribosomal RNA genes. Furthermore, this chloroplast genome contains six pseudogenes, including a pair of *ndh*B a pair of *ndh*D, one *ndh*C, and one *ndh*K. The phylogenetic relationship within the genus *Corydalis* was inferred with the maximum-likelihood method, and the result showed that *C. impatiens* was most closely related to *C. conspersa*.

*Corydalis impatiens* (Pall.) Fisch., 1821. (Papaveraceae) is an annual herb of the family Fumariaceae, and it grows in foothills, crevices in rocks, shady slopes under forests and thickets. *C. impatiens* also known as Pa Xia Ga, is a Tibetan medical herb (Pan et al. 2019). This traditional medicine can reduce pain, treat skin injuries, cure hepatitis, and benefit the circulatory system (Niu et al. 2013). A clear understanding of chloroplast genome information not only contributes to species identification, phylogenetic analysis and molecular breeding, but also provides a molecular basis for important cash crops, horticultural variety improvement and conservation of rare and endangered plants (Moore et al. 2010; Nie et al. 2012; Shaw et al. 2014; Zhu et al. 2021). At present, although there are some studies on the medicinal value of *C. impatiens* (Niu et al. 2013), there are few studies on its genetics. Here, the chloroplast genome of *C. impatiens* was sequenced, and a phylogenetic analysis of *C. impatiens* and its allies was carried out.

Fresh young leaves of *C. impatiens* were collected from Huangzhong County, Qinghai Province, China (36.27°N, 101.68°E). The voucher specimen (specimen accession number: LQE-2020-070) was deposited in the Specimen Room of the Tibetan Medicine Research Center of Qinghai University (https://zyxy.qhu.edu.cn/jgsz/jxkysw/zyyyjzx/index.htm, Qien Li, qienli@qhu.edu.cn). Total DNA was extracted from Silica gel dried young leaves with a Plant Genomic DNA Kit (DP305, TIANGEN Biotech (Beijing) Co., Ltd., Beijing, China). Qualified DNA fragmentation was carried out by Ultrasonic Processor, the length of insert fragment was approximately 350 bp. Then terminal repair, add base A, add sequence adapter, purification, PCR amplification were implemented to complete the 350 bp library preparation. Whole-genome sequencing was conducted by Novogene Co., Ltd. (Tianjin, China) with the Illumina NovaSeq 6000 Sequencing System (Illumina, San Diego, CA). Approximately, 15.8 GB of clean data were generated. SPAdes version 3.10.1 (Bankevich et al. 2012) and SSPACE version 2.0 (Boetzer et al. 2011) were used to assemble the chloroplast genome with default settings. This process does not use a reference genome. The assembled complete chloroplast genome was annotated with CPGAVAS2 (http://www.herbalgenomics.org/cpgavas2) (Shi et al. 2019) and the sequence coordinates for the genes were verified by BLAST search against the *C. inopinata* (GenBank accession number: NC_052866.1) chloroplast genome.

The assembled *C. impatiens* chloroplast genome was 197,317 bp in length with a GC content of 40.68%. As seen in most chloroplast genomes, this complete genome showed a typical quadripartite structure comprising a pair of inverted repeats (IRs 52,211 bp), one large single-copy (LSC, 89,790 bp) region, and one small single-copy (SSC, 3,105 bp). The chloroplast genome of *C. impatiens* encodes a total of 127 functional genes, including 81 protein-coding, 38 transfer RNA, and eight ribosomal RNA genes, accounting for 63.78%, 29.92%, and 6.30% of all annotated functional genes, respectively. Moreover, this chloroplast genome contains six pseudogenes, namely a pair of *ndhB*, a pair of *ndhD*, one *ndhC*, and one *ndhK*.

The phylogenetic relationship within the genus *Corydalis* was inferred with the maximum-likelihood (ML) method based on the Tamura–Nei model (Tamura and Nei,^1993^), with *Lamprocapnos spectabilis* used as the out-group. In particular, a total of 16 complete chloroplast sequences of *C. impatiens* and its allies were aligned by MAFFT version 7.475 (Katoh and Standley 2013) using default settings. The percentage of trees in which the associated taxa clustered together is shown next to the branches. Initial tree(s) for the heuristic search were obtained automatically by applying Neighbor-Joining and BioNJ algorithms to a matrix of pairwise distances estimated using the maximum composite likelihood approach, and then topology with superior log likelihood value was selected. The tree was drawn to scale, with branch lengths indicating substitutions per site. All positions containing gaps and missing data were eliminated. There were a total of 90,759 positions in the final dataset. Phylogenies were generated using the MEGA7 program based on general time-reversible (GTR)/GTR + I+G nucleotide substitution models of ML (Kumar et al. 2016), and the strengths of phylogenies were evaluated by resampling with 200 bootstrap replications. The phylogenetic analysis showed that *C. impatiens* was most closely related to *C. conspersa* (Figure 1).

**Figure 1.**
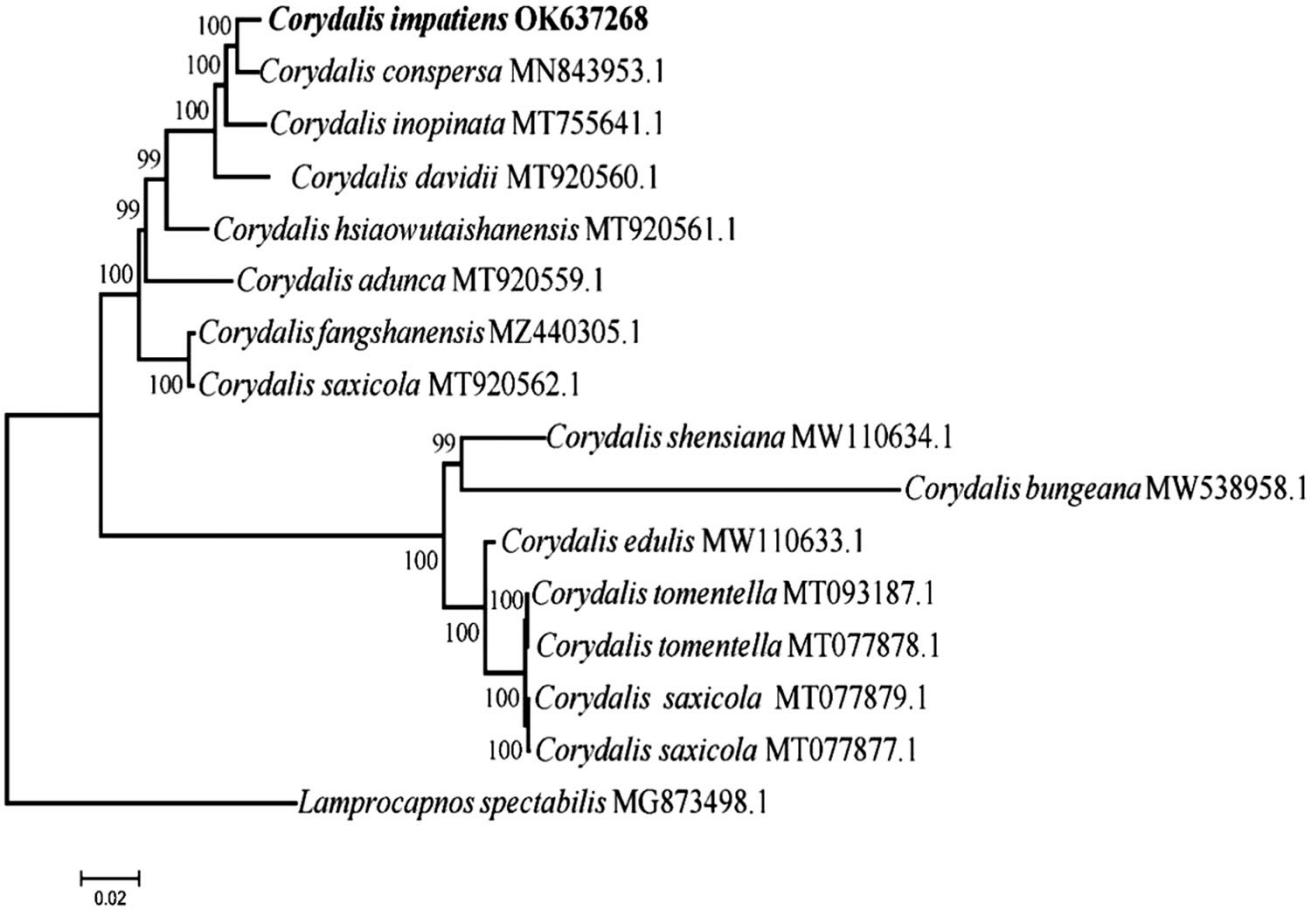
Maximum-likelihood (ML) tree of 16 species based on the complete chloroplast sequences. Numbers above branches are bootstrap percentages (based on 500 replicates).

The present study enriched the understanding of the maternal genetic information of *C. impatiens*, and provided additional data for reconstructing species relationships within the genus *Corydalis.*

## Ethical approval

The materials used in this study are not included in the IUCN Red List of Threatened Species or the List of State-protected Plant Species, and the sampling site is not located in any protected area. The field study and laboratory study were conducted in accordance with guidelines provided by Qinghai University.

## Author contributions

Digao Wan and Qien Li were involved in the conception and design; Haijuan Bao, Qupei Danzeng, and Xiao Guo analyzed and interpreted the data; Digao Wan drafted the paper; Xiao Guo and Qien Li revised it critically for intellectual content. All authors approved the final version to be published, and agreed to be accountable for all aspects of the work.

## Data Availability

The genome sequence data obtained in this study are openly available in GenBank of NCBI at https://www.ncbi.nlm.nih.gov/ under the accession number OK637268. The associated BioProject, SRA, and Bio-Sample numbers are PRJNA776704, SRX12869145, and SAMN22814974, respectively.
